# Supplementary material for: Genome-Wide Dissection of Novel QTLs and Genes Associated with Weed Competitiveness in Early-Backcross Selective Introgression-Breeding Populations of Rice (Oryza sativa L.)
Source: Biology (Basel). 2025 Apr 13;14(4):413. doi: 10.3390/biology14040413 (PMC12025310; doi:10.3390/biology14040413)
Supplement: Supplementary file 1 [file biology-14-00413-s001.zip › Supplemental Tables.pdf]

Supplemental Tables

Supplemental Table S1. Early seed germination (ESG) and early seedling vigor (ESV) traits measured.

NO.	TRAIT	DESCRIPTION OF TRAIT
Early Seed Germination		
1	Second-day Germination Count	Number of seeds germinated after 2 days
2	Seventh-day Germination Count	Number of seeds germinated after 7 days
3	Germination Rate	Second-day Germination Count divided by Seventh-day Germination Count
4	Coleoptile Length	Length from collar region until tip of topmost leaf
5	Radicle Length	Length from collar region until tip of longest root
6	Total Dry Weight of Germinated Seeds	Total dry weight of all seeds that germinated
7	Average Dry Weight of Germinated Seeds	Total dry weight of all seeds that germinated divided by number of seeds that germinated
8	Seed Vigor Index	Product of germination rate and total dry weight of seeds that germinated
Early Seedling Vigor		
1	Plant Height at 14 DAS	Measurement from ground level until tip of longest leaf at 14 DAS
2	Plant Height at 21 DAS	Measurement from ground level until tip of longest leaf at 21 DAS
3	Plant Height at 28 DAS	Measurement from ground level until tip of longest leaf at 28 DAS
4	Leaf Count at 14 DAS	Number of leaves at 14 DAS
5	Leaf Count at 21 DAS	Number of leaves at 21 DAS
6	Leaf Count at 28 DAS	Number of leaves at 28 DAS
7	Tiller Number at 28 DAS	Number of tillers at 28 DAS
8	Seedling Vigor Index at 28 DAS	100% germination rate multiplied by total dry weight
9	Shoot Dry Weight at 28 DAS	Dry weight of leaves after drying at 70°C for 5 days
10	Root Dry Weight at 28 DAS	Dry weight of root after drying at 70°C for 5 days
11	Total Dry Weight at 28 DAS	Sum of leaf dry weight and root dry weight
12	Root Length at 28 DAS	Length from collar region until tip of longest root

Supplemental Table S2 Selected 15 well-performing early backcross selective-introgression lines (EB-SILs) based on key early seed germination (ESG) traits (Common recipient parent is WTR-1).

No.	Designation	Donor Parents
1	GSR IR2-1-R14-L1-R2	Haoannong
2	GSR IR2-1-Y10-Y1-L2	Haoannong
3	GSR IR2-1-Y12-L1-L2	Haoannong
4	GSR IR2-1-Y12-L1-R2	Haoannong
5	GSR IR2-1-Y13-L1-L2	Haoannong
6	GSR IR2-1-Y14-L1-R3	Haoannong
7	GSR IR2-1-Y14-Y1-R2	Haoannong
8	GSR IR2-1-Y9-Y1-Y2	Haoannong
9	GSR IR2-4-L12-S2-L2	Cheng Hui 448
10	GSR IR2-4-L5-L2-L2	Cheng Hui 448
11	GSR IR2-4-L6-S1-L2	Cheng Hui 448
12	GSR IR2-4-L9-L2-L2	Cheng Hui 448
13	GSR IR2-4-R8-S1-Y2	Cheng Hui 448
14	GSR IR2-4-Y10-L1-L2	Cheng Hui 448
15	GSR IR2-6-R3-S2-R2	Y-134

Supplemental Table S3 Selected 15 best performing early backcross selective-introgression lines (EB-SILs) based on key early seedling vigor (ESV) traits. (Common recipient parent is WTR-1).

No.	Designation	Donor Parent
1	GSR IR2-1-L1-S1-Y2	Haoannong
2	GSR IR2-1-L1-Y1-L2	Haoannong
3	GSR IR2-1-L13-DT1-R2	Haoannong
4	GSR IR2-1-Y16-S1-L2	Haoannong
5	GSR IR2-4-R2-L1-L2	Cheng Hui 448
6	GSR IR2-4-R2-S1-L2	Cheng Hui 448
7	GSR IR2-4-R6-S1-L2	Cheng Hui 448
8	GSR IR2-4-R6-S1-R2	Cheng Hui 448
9	GSR IR2-4-Y6-L1-L2	Cheng Hui 448
10	GSR IR2-6-R4-S4-R2	Y-134
11	GSR IR2-6-R8-L1-L2	Y-134
12	GSR IR2-6-R8-L1-L3	Y-134
13	GSR IR2-6-R8-L1-Y2	Y-134
14	GSR IR2-6-R8-Y1-L2	Y-134
15	GSR IR2-6-R8-Y1-R2	Y-134

Supplemental Table S4. Possible candidate genes identified from the 18 quantitative trait loci (QTLs) for weed competitiveness of rice.

No.	Chr.	CDS Co (5'-3')		Annotation of the gene	Locus
		Start	End		
Early Seed Germination Traits					
1	2	34408089	34413407	GATA zinc finger domain containing protein, expressed	LOC_Os02g56250
2	2	8516600	8518271	late embryogenesis abundant domain-containing protein, putative, expressed	LOC_Os02g15250
3	2	8582543	8584541	no apical meristem protein, putative, expressed	LOC_Os02g15340
4	2	8590287	8593925	dof zinc finger domain containing protein, putative, expressed	LOC_Os02g15350
5	2	30528645	30531120	ZIM motif family protein, expressed	LOC_Os02g49970
6	2	30674776	30678659	glutamine synthetase, catalytic domain containing protein, expressed	LOC_Os02g50240
7	2	30729063	30736459	RNA-dependent RNA polymerase, putative, expressed	LOC_Os02g50330
8	6	1629717	1633629	aminotransferase, classes I and II, domain containing protein, expressed	LOC_Os06g03990
9	6	1635096	1636029	peptidyl-prolyl cis-trans isomerase, putative, expressed	LOC_Os06g04000
10	6	1674045	1677406	pyridoxal-dependent decarboxylase protein, putative, expressed	LOC_Os06g04070
11	6	1765622	1770656	starch synthase, putative, expressed	LOC_Os06g04200
12	12	5756436	5760088	glutathione S-transferase, putative, expressed	LOC_Os12g10720
13	12	5760209	5763724	glutathione S-transferase, putative, expressed	LOC_Os12g10730
14	12	6916185	6918603	NADP-dependent oxidoreductase, putative, expressed	LOC_Os12g12580
Early Seedling Vigor					
15	1	42281272	42281973	abscisic stress-ripening, putative, expressed	LOC_Os01g72900
16	1	42282832	42283632	abscisic stress-ripening, putative, expressed	LOC_Os01g72910
17	1	42460555	42462334	abscisic stress-ripening, putative, expressed	LOC_Os01g73250
18	5	28572408	28575233	histone-like transcription factor and archaeal histone, putative, expressed	LOC_Os05g49780
19	9	14617482	14619069	No apical meristem protein, putative, expressed	LOC_Os09g24560
20	9	14775451	14776791	MYB family transcription factor, putative, expressed	LOC_Os09g24800
21	9	14793742	14794460	ZF-HD protein dimerization region containing protein, expressed	LOC_Os09g24810
22	9	14812099	14816389	ZF-HD protein dimerization region containing protein, expressed	LOC_Os09g24820
23	9	14617482	14619069	No apical meristem protein, putative, expressed	LOC_Os09g24560
24	3	35953611	35955166	brevis radix, putative, expressed	LOC_Os03g63650
25	10	18051860	18056913	auxin response factor 18, putative, expressed	LOC_Os10g33940
26	10	18085536	18092500	START domain containing protein, expressed	LOC_Os10g33960
27	10	18145792	18149595	glutathione S-transferase, putative, expressed	LOC_Os10g34020
28	10	18360484	18372591	Dicer, putative, expressed	LOC_Os10g34430
