# Supplementary figures and images for: Genome-Wide Dissection of Novel QTLs and Genes Associated with Weed Competitiveness in Early-Backcross Selective Introgression-Breeding Populations of Rice (Oryza sativa L.)
Source: Biology (Basel). 2025 Apr 13;14(4):413. doi: 10.3390/biology14040413 (PMC12025310; doi:10.3390/biology14040413)

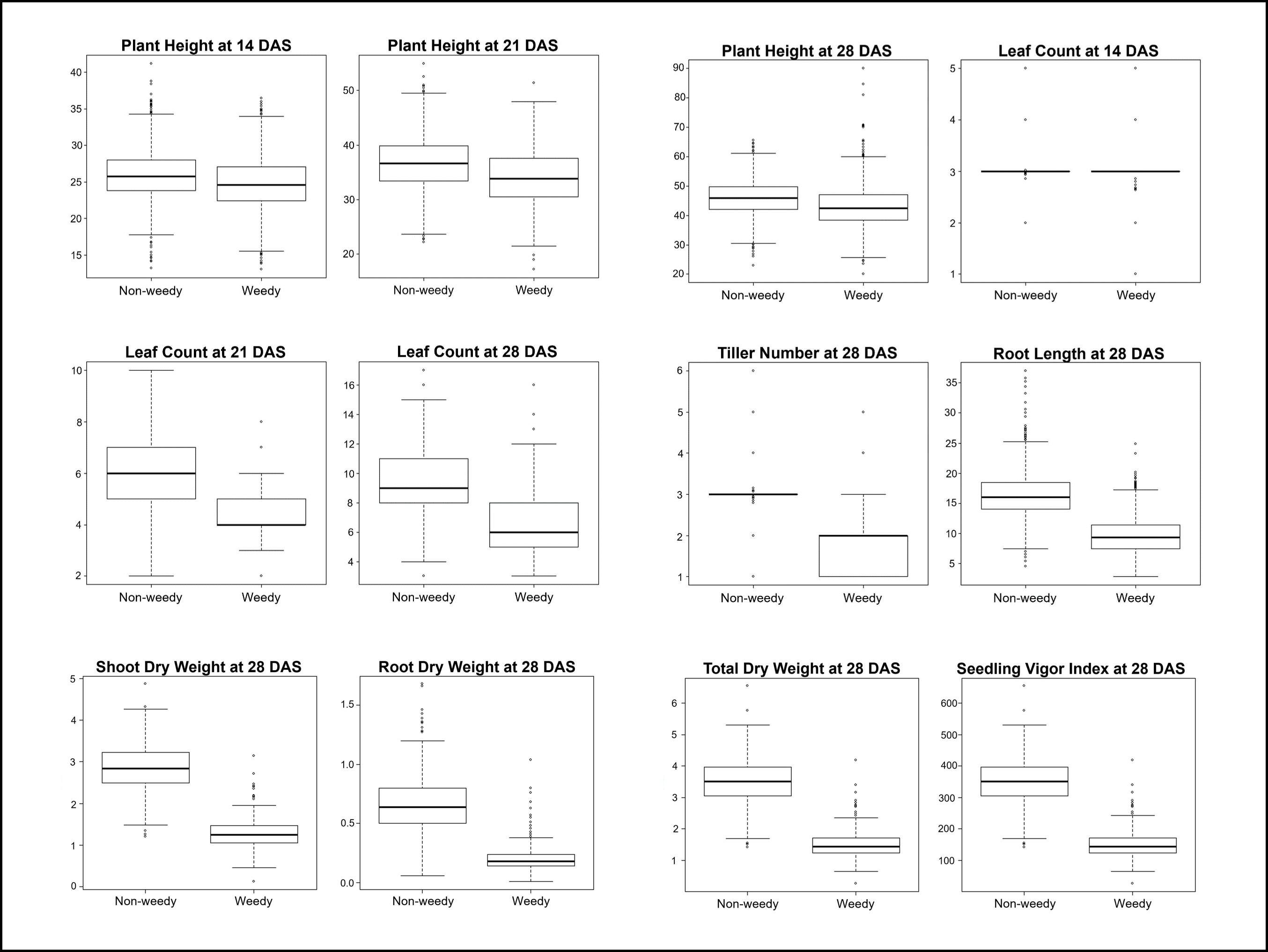

Supplement: Supplementary file 1 [file biology-14-00413-s001.zip › Supplementary Figure 1.tif]

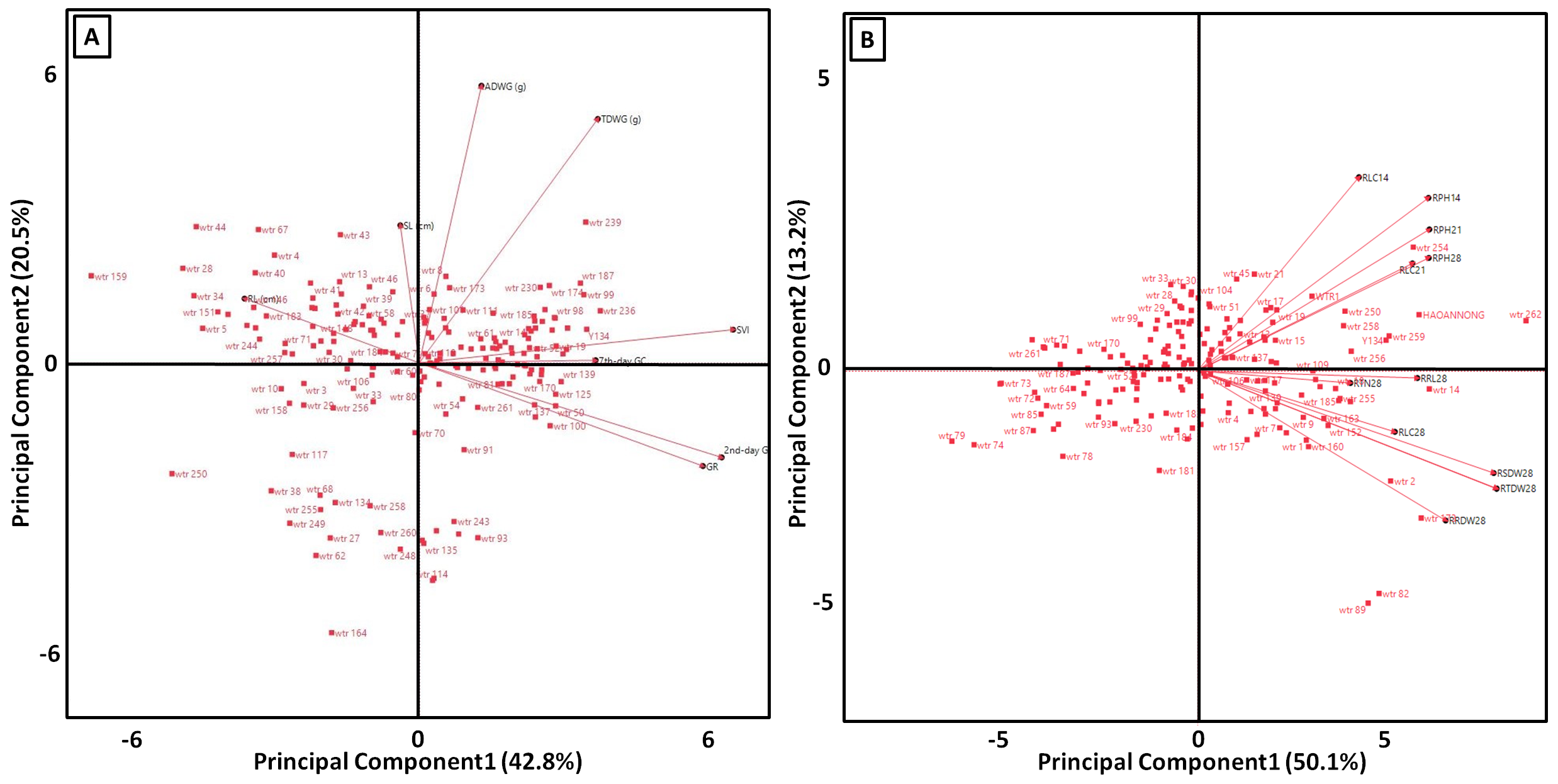

Supplement: Supplementary file 1 [file biology-14-00413-s001.zip › Supplementary Figure 2.tif]
